# Anti-jamming communication for body area network using chaotic frequency hopping

**DOI:** 10.1049/htl.2017.0041

**Published:** 2017-11-13

**Authors:** Balamurugan Gopalakrishnan, Marcharla Anjaneyulu Bhagyaveni

**Affiliations:** 1 Department of Electronics Engineering, MIT Campus, Anna University, Chennai, Tamilnadu, India; 2 Department of Electronics and Communication Engineering, College of Engineering Guindy, Anna University, Chennai, Tamilnadu, India

**Keywords:** body area networks, chaotic communication, security of data, frequency hop communication, body sensor networks, telemedicine, jamming, error reduction, information transmission, chaos-based hop selection, channel selection, throughput degradation, wireless networks, blocking communication, wireless communication, healthcare applications, security, patient reliable communication, chaotic frequency hopping, body area network, anti-jamming communication

## Abstract

The healthcare industries research trends focus on patient reliable communication and security is a paramount requirement of healthcare applications. Jamming in wireless communication medium has become a major research issue due to the ease of blocking communication in wireless networks and throughput degradation. The most commonly used technique to overcome jamming is frequency hopping (FH). However, in traditional FH pre-sharing of key for channel selection and a high-throughput overhead is required. So to overcome this pre-sharing of key and to increase the security chaotic frequency hopping (CFH) has been proposed. The design of chaos-based hop selection is a new development that offers improved performance in transmission of information without pre-shared key and also increases the security. The authors analysed the performance of proposed CFH system under different reactive jamming durations. The percentage of error reduction by the reactive jamming for jamming duration 0.01 and 0.05 s for FH and CFH is 55.03 and 84.24%, respectively. The obtained result shows that CFH is more secure and difficult to jam by the reactive jammer.

## 1 Introduction

**Fig. 1:**
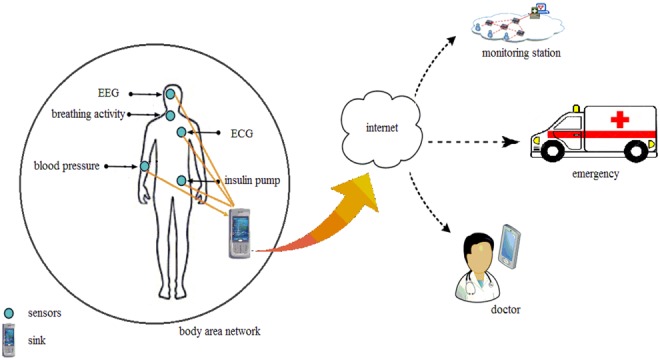
Wireless body area network architecture

Wireless body area network [1] (WBAN) consists of discrete group of independent sensor nodes placed in and around human body to monitor physiological information such as electrocardiogram, electroencephalogram, blood pressure, blood glucose, respiration rate levels. The crucial sensor monitors the vital physiological parameters to diagnose the disease and significantly monitors the health status. The sensor nodes in BAN typically form star topology; all the nodes can communicate with a coordinator node or sink or hub, usually a personal digital assistant (PDA) device. The collected information by the PDA or hub processes the information and transmits it to the medical server over a shared medium. Fig. 1 shows the placement of sensors and architecture of WBAN.

Due to the broadcast nature of wireless communication, wireless networks are vulnerable to both intentional and un-intentional jamming attacks. The jamming signal disturbs network communications between legitimate users and results in throughput degradation, link failure. Apart from different jamming attacks, reactive jamming [2] is one of the most effective jamming attacks. A reactive jammer continuously listens for the channel activities, and emits jamming signals whenever it detects activities, otherwise it stays quiet when the sender is idle. Reactive jamming is regarded as one of the most effective, stealthy and energy efficient jamming strategies.

The traditional FHSS [3] does not provide higher security against jammer because it uses fixed hopping pattern. If the transmitter is hopping the frequency at a faster rate, it results reduction in throughput because of channel switching. Also both the transmitter and receiver should pre-share the fixed hopping sequence in the presence of jammer. So to overcome the drawbacks we propose chaotic frequency hopping (CFH) where the hopping sequence is selected by using chaotic [4] map signal. The chaotic signal exhibits chaotic behaviour due to its properties; it will completely confuse the reactive jammer to choose the correct hopping channel.

The main objective of this work is to study the effectiveness of CFH techniques against reactive jamming attacks. First, we developed CFH technique with no pre-shared secret keys as in traditional FHSS. Second, we analysed the BER performance under reactive jamming attack with traditional frequency hopping (FH) and uncoordinated FH [5]. The obtained results show that CFH technique work effectively well against reactive jamming attacks

In rest of this paper, Section 2, we discussed research work carried to overcome jamming attacks by using FH techniques. In Section 3, we described working principle of CFH. Matlab simulation was performed and related results discussed in Section 4. Finally, Section 5 briefly summarises our proposed work and outlines possible directions of future work.

## 2 Related works

Recently, there is a series of research work relating to anti-jamming in wireless communication. Most previous studies employ FH to avoid jammers. Based on literature review, we broadly classified anti-jamming techniques into two major classes as proactive FH and reactive FH which are commonly used techniques to overcome jamming. We briefly reviewed few anti-jamming techniques.

In traditional FHSS [3], transmitter transmits radio signals by rapidly switching a carrier among many frequency channels, where the channel switching is based on spreading codes that should be pre-shared between transmitter and receiver. If the jammer knows the post knowledge of the channels, then it has an ability to determine the next channel by means of a random guess. Based on channel switching frequency hop is classified into two types: proactive channel hopping and reactive channel hopping.

In proactive channel hopping technique, the transmitter dynamically switches to new frequency independent to channel conditions. Popper *et al.* [5] discuss uncoordinated frequency-hopping (UFH) technique to overcome anti-jamming communication and key establishment in presence of jammer over wireless medium. This technique provides low throughput and latency increases. Navda *et al.* [6] implement a frequency-hopping protocol with pseudorandom channel switching. They compute the optimal frequency-hopping parameters, assuming that the jammer is aware of the procedure followed. The author in [7] developed a novel FH spread spectrum technique using random pattern table. In all nodes, twenty hopping patterns along with seed values are stored. Each pattern uses six hops and these hops changes adaptively at different instant time. The drawback of this technique is that nodes need to store lot of seed values to attain lengthy non-repetitive hopping sequence. Zhou *et al.* [8] proposed adaptive UFH an online learning algorithm for adaptive channel access of wireless communications in unknown environments. It dynamically selects a subset of channels to maximise its accumulated data rates over time.

In reactive channel hopping technique, the transmitter switches to new channel only when the current channel is jammed. Due to this hopping rate is minimised when compared with proactive techniques. Xu *et al.* [9] designed an algorithm in which a sensor node adaptively changes the working channel when it detects strong jamming signals in the current channel. Honeynode [10] identifies the jammer with the help of dummy frequency and the channel switching occurs if dummy frequency detects jammer. Yadong *et al.* [11] proposed reliable aware frequency-hopping algorithm to increase the network reliability. In this technique, the frequency is switched to a new frequency channel when the packet delivery ratio becomes less than the given threshold. However, when this method is applied to burst nature of wireless channel then network reliability degrades.

The above literature review states that frequency hop is not effective against reactive jamming attack. This motivates our research work to focus towards the chaotic FH technique. The chaotic [4] signals are dynamic systems and due to its erratic behaviour it is very difficult for the reactive jammer to guess the code sequence. Mansour *et al.* [12] proposed cross-coupled chaotic matched FH to mitigate partial band noise jamming. In [13], the author presented a comprehensive survey of different chaos-based digital communication systems. The above works motivate our research to exploit on chaotic signal to use in spread spectrum. In our CFH approach, channel selection is performed by using chaotic map. The proposed technique aims to increase the security of the network without pre-shared key. In Section 3, we discussed the proposed CFH technique in detail.

## 3 Proposed work

In anti-jamming communication, we present a novel FHSS communication system by using chaotic signal. The chaotic signal exhibits chaotic behaviour, due to its properties it will completely confuse the eavesdropper. By using the chaotic signal, the selection of hop for transmitting and recovering the information is carried by chaos signal. The design of chaos-based hop selection is a new development that offers improved performance in transmission of information without pre-shared key and also increases the security.

### 3.1 Chaotic FHSS transmitter and receiver block

**Fig. 2:**
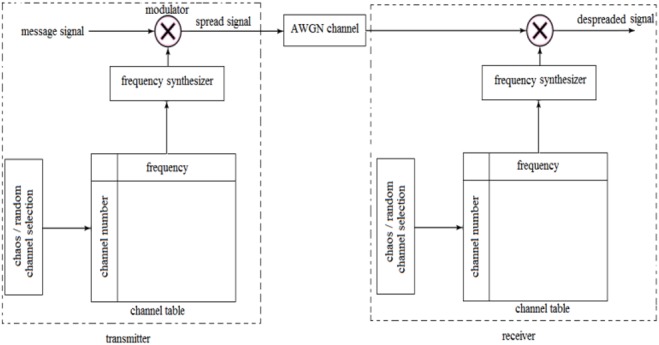
Chaotic frequency hop spread spectrum

In CFH, channel selection is performed in two phases: random channel selection phase and chaos channel selection phase. In random channel selection phase, the transmitter and receiver do not rely on secret channels but instead transmit and listen on randomly selected frequency. At particular instant the sender and receiver channel frequency coincide with each other. In that time instant transmitter exchanges initial seed value of chaotic signal to the receiver. In channel selection phase, the obtained initial seed value is applied to the chaotic map to generate chaotic values. These chaotic values are used for selecting frequency hops for data transmission. Fig. 2 shows transmitter and receiver process in CFHSS.

### 3.2 Channel table

The frequency band used is 2.4 GHz and it consists of 79 RF channels and each channel has 1 MHz bandwidth. The frequency band starts at 2400 MHz and ends at 2483.5 MHz. Let the symbol $\Delta f$ denotes the frequency number and its range is $\Delta f = \lcub 0\comma \; 1\comma \; 2\comma \; 3\comma \; 4 \ldots \comma \; \lpar M - 1\rpar \rcub $, where *M* is the number of channels. The centre frequency $f_{\Delta f}$ of the channel $\Delta f$ is given by

(1) $$f_{\Delta f} = \left({2402 + \Delta f} \right)\, {\rm MHz}\eqno\lpar 1\rpar $$

The frequency spectrum is shown in Table 1 for each channel. The FH is performed by switching the carrier with different frequencies.

**Table 1:** Channel table

Channel number $\Delta f$	Frequency spectrum, MHz
0	2401.5–2402.5
1	2402.5–2403.5
2	2403.5–2404.5
.	.
.	.
78	2479.5–2480.5

### 3.3 Channel selection

To establish communication between two legitimate users, nodes should operate in two stages. First the random channel selection and followed by chaos channel selection.

#### 3.3.1. Random channel selection

The transmitter randomly selects the channel from a predefined set of channel frequency $\lpar \Delta f\rpar $. Where *n* indicates the number of channels

(2) $$\Delta f = \lcub \Delta f_1\comma \; \Delta f_2\comma \; \Delta f_3\comma \; \ldots \comma \; \Delta f_n\rcub \eqno\lpar 2\rpar $$

**Fig. 3:**
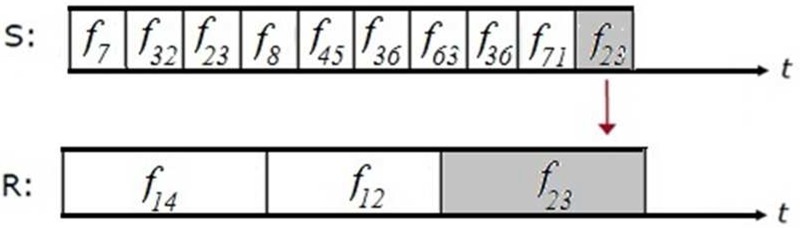
Timing diagram of random channel selection

Similarly receiver also hops randomly from a predefined set of channels $\left({\Delta f} \right)$ but it hops slowly. In Fig. 3, the sender (S) and receiver (R) meet occasionally with same hop. On that time duration sender exchanges chaotic initial value $\lpar x_{0\; }\rpar $ and bifurcation parameter $\lpar r\rpar $ to the receiver.

The sender hops the channel randomly at faster rate when compared with receiver. The exchanged chaotic initial seed value is used in chaos code generator which selects the forthcoming channels for information transmission.

#### 3.3.2. Chaos channel selection

Chaotic sequences are generated by discrete chaotic functions. The most commonly used chaotic maps are tent (triangular), logistic (quadratic), bernoulli (saw tooth) maps

(3) $${\rm tent}\, \, {\rm map}\comma \; \quad x_{i + 1} = 2r\left({1 - \left\vert {x_i} \right\vert } \right)- 1\eqno\lpar 3\rpar $$

(4) $${\rm logistic}\, \, {\rm map}\comma \; \quad x_{i + 1} = \left({\displaystyle{r \over 2}} \right)\left({1 - x_i^2 } \right)- 1\eqno\lpar 4\rpar $$

(5) $$\eqalign{{\rm bernoulli}\, \, {\rm map}\comma \; \quad x_{i + 1} & = rx_i + 1\comma \; \quad x_i \lt 0 \cr x_{i + 1} & = rx_i - 1\comma \; \quad x_i \gt 0} \eqno\lpar 5\rpar $$

where *r* is the bifurcation parameter and $x_i$ is the state of discrete-time dynamic system. The bifurcation parameters are chosen such that the dynamics of the maps fall into their chaotic regime. The chaos maps is better than other digital communication system, due to its characteristics such as easy implementation, a-periodic, not easy to predict, broadband and sensitive to initial conditions. The transmitter and receiver exchange chaotic initial value $\lpar x_{0}\rpar $ and bifurcation parameter $\lpar r\rpar $.

By using initial parameters as an input to any chaotic map (3)–(5), both transmitter and receiver generate chaotic signal. By iterating the chaotic equation, different discrete values $x_{i + 1}$ are obtained and the values are in the range between $x_{i + 1} \in \lsqb - 1\comma \; + 1\rsqb .$The obtained discrete chaotic value $x_i$ is converted to chaotic integer value $C_i$ by using (6) where $\vartheta $ is whole number and it should be $\vartheta \gt 2$

(6) $$C_i = {\mathop{\rm int}} \lsqb \lpar x_i \times 10^\vartheta \rpar \rsqb \eqno\lpar 6\rpar $$

The modulus operation is performed for (6) to obtain the chaotic index $\lpar I_i\rpar $ value to bring the index value within the range of total number of frequency channels (*N*)

(7) $$I_i = C_i\bmod N\eqno\lpar 7\rpar $$

The transmitter and receiver selects the frequency hop based on the generated chaotic index $\lpar I_i\rpar $ values. The intended receiver performance is almost similar to conventional FHSS but chaotic-based FHSS can result in higher probability of error for intruders that do not know the initial value parameters.

### 3.4 Data transmission using chaotic frequency

The data transmission between legitimate nodes starts in this phase. The initial value of chaotic signal which was exchanged between two legitimate nodes in random channel phase is used for selecting chaos channel. Depending on the initial value, the chaos code generator using (3)–(5) is used to generate chaotic iterative peak values. The chaotic peak $\lpar x_i\rpar $ values which were in fractional number $\lpar C_f\rpar $ are converted to a whole number $\lpar C_i\rpar $ and it is represented as chaotic index. The chaos channel is selected from $\lpar C_i\rpar $ by using modular operator. Table 2 shows the chaotic channel selection for the chaotic initial value $\lpar x_{0\; }\rpar $ as 0.6 and bifurcation parameter $\lpar r\rpar $ as 0.89 for chaotic tent map.

**Table 2:** Chaotic channel selection

Chaotic map peaks $\lpar x_i\rpar $	Chaotic fractional value $\lpar C_f\rpar $	Chaotic integer value $\lpar C_i\rpar $	Channel no $\lpar I_i\rpar $
−0.2440	−244	−244	72
0.4288	428.8	429	34
0.0795	79.49	80	1
0.7398	739.75	740	29
−0.5081	−508.14	−508	45
.	.	.	.
.	.	.	.

Fig. 2 shows CFHSS transmitter and receiver block. In random channel selection phase, both transmitter and receiver share the initial chaotic seed value. The obtained seed value is used in chaotic channel phase to select the chaotic channel shown in Table 2. The frequency synthesizer generates a range of frequencies corresponding to the selected chaotic channel number and then it is modulated with the message signal and finally it is transmitted to the AWGN channel. Similarly at the receiver by using same chaotic value it will try to de-spread the message signal.

## 4 Simulation results

In this section, employing MATLAB computer simulation the performance of chaotic frequency hop was evaluated. A comparison of bit error rate (BER) performance, security analysis under reactive jamming attack for chaos-based FH technique is compared with other spreading techniques like conventional FHSS, CFHSS and UFHSS are presented. From the obtained results, it is found that our proposed technique overcomes reactive jamming and provides more secured communication than other techniques.

### 4.1 BER analysis

The BER performance of the proposed communication system with the channel noise being AWGN has been evaluated using Matlab. The simulated BERs are calculated as the total number of error bits divided by the total number of transmitted bits. The BER performance of CFHSS is compared with traditional FHSS and UFHSS techniques. The BER performance analysis is tested by transmitting 10^5^ message bits and in each hop 10 symbols are transmitted.

**Fig. 4:**
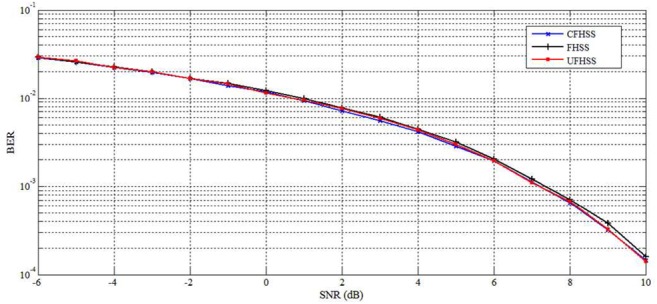
BER performances analysis for FHSS, CFHSS and UFHSS

Fig. 4 shows that the BER performance of proposed CFHSS, traditional FHSS and uncoordinated FHSS technique. The result implies that traditional FHSS, chaotic FHSS and uncoordinated FHSS show similar BER performance under different SNR values. For SNR 5 dB, the BER value for traditional FHSS, CFHSS and UFHSS techniques gives 0.003188, 0.002832 and 0.003014, respectively.

### 4.2 Security analysis under reactive jamming attack

**Fig. 5:**
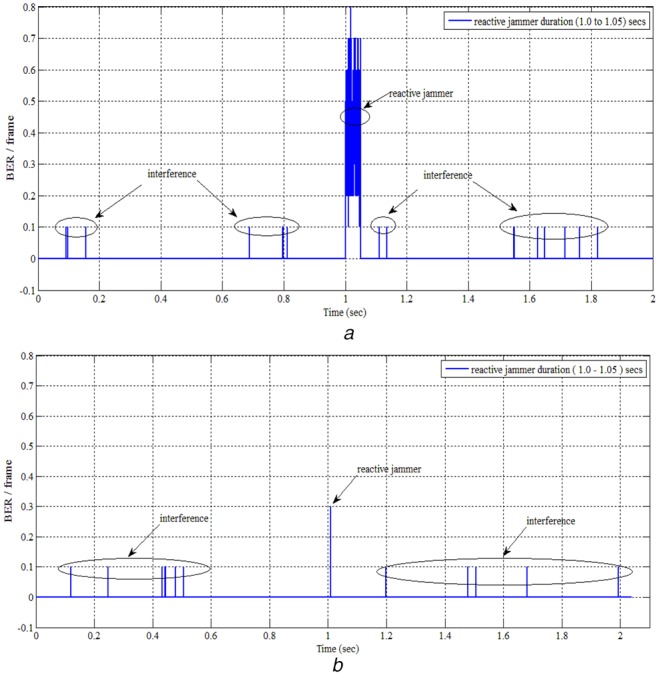
BER Performance against reactive jamming duration of 0.05 s *a* FHSS *b* CFHSS

The reactive jammer [2] stays quiet when the channel is idle, but starts transmitting a radio signal as soon as it senses activity on the channel. One advantage of a reactive jammer is that it is harder to detect. Figs. 5*a* and *b* show reactive jamming for a period of 0.05 s jamming the transmitting channel under FHSS and CFHSS, respectively. The reactive jammer is switched on and off between 1.0 and 1.05 s. The simulation is performed by transmitting 10^5^ message bits and in each hop 10 symbols are transmitted with SNR value 10 dB. Since the fixed hopping pattern is known to the jammer, it is easy to jam the communication continuously. In Fig. 5*a*, due to the reactive jammer a high BER occurs, whereas in other time instants BER occurs due to channel interference.

Similarly in CFHSS, reactive jammer is turned on for duration between 1.0 and 1.05 s. Since in CFHSS, the channel is selected based on chaotic maps under every instant hopping frequency is selected based on previous chaotic value. The reactive jammer tries to find the hopping pattern used at every instant. If the reactive jammer is successful in knowing the pattern used at a particular instant, then it cannot continuously perform jamming as the pattern keeps changing at different instants.

In Fig. 5*b*, the reactive jammer is successful only for a particular instant of time and since the pattern changes based on chaotic value, it is very difficult for the jammer to guess the hopping pattern used by the legitimate node. This makes the proposed technique effective for applications dealing with sensitive data.

### 4.3 BER performance analysis under reactive jammer

**Fig. 6:**
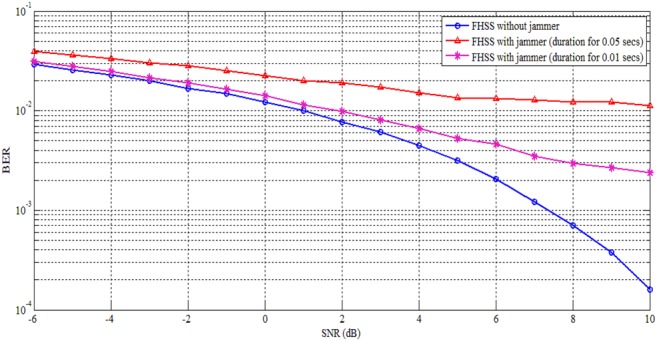
FHSS analysis with and without reactive jammer

The BER performance analyses against reactive is tested by transmitting 10^5^ message bits and in each hop 10 symbols are transmitted. The channel noise being AWGN has been evaluated practically. Fig. 6 shows the FHSS with and without reactive jammer under different jamming duration.

**Fig. 7:**
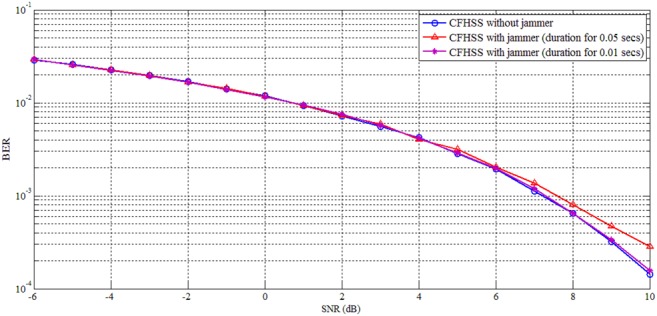
CFHSS analysis with and without reactive jammer

From Fig. 6, it is inferred that in FHSS when reactive jammer is switched on, more number of bits get jammed; therefore BER increases and it is based on jamming duration. For SNR 6 dB, the BER for FHSS without reactive jammer gives 0.002044, whereas FHSS with reactive jammer shows 0.0046 and 0.01338 for jamming duration 0.01 and 0.05 s, respectively. The BER increases because FHSS uses fixed hopping pattern. Fig. 7 shows the CFHSS with and without reactive jammer under different jamming duration. For SNR 6 dB, the BER for CFHSS without reactive jammer gives 0.001954, whereas CFHSS with reactive jammer shows 0.00203 and 0.00211 for jamming duration 0.01 and 0.05 s, respectively.

The percentage of error reduction by the reactive jamming for jamming duration 0.01 and 0.05 s for FHSS and CFHSS is 55.03 and 84.24%, respectively. The jamming effectiveness by increasing the jamming duration for 0.05 from 0.01 s is ascertained as 72% under the FHSS, whereas it is only 3.4% in the proposed CFHSS technique. The jamming effectiveness in FHSS increases because it uses fixed hopping pattern for channel selection, whereas in CFHSS technique channel is selected based on chaotic map. The above results demonstrate that CFHSS is more secure and effective in defending against reactive jamming attack because hopping pattern depends on chaotic map. Since for every instant hopping frequency changes, it is very difficult for the reactive jammer to jam. Even if the attacker is successful in knowing the pattern used at a particular instant, jammer cannot continuously jam as the pattern keeps changing at different instants.

### 4.4 Computation time

The computation complexity is analysed by transmitting 10^3^ bits for FHSS, UFHSS and CFHSS techniques and it is shown in Table 3. The computation time for FHSS is 0.0802 s which is very low compared with other techniques because of fixed hopping pattern but the chance of reactive jamming is high, whereas the computation time for UFHSS and CFHSS technique is 0.1681 and 0.0825 s, respectively. The UFHSS computation time is high because each time the sender randomly chooses the communication channels for the transmission of message. However, in CFHSS random channel selection occurs only at the initial stage to exchange chaotic initial parameters. Thereafter the forthcoming channels are selected by using chaotic map for message transmission. So computation time is less when compared with UFHSS and provides better security than FHSS.

**Table 3:** Computation complexity

Techniques	FHSS	UFHSS	CFHSS
computation time (s)	0.0802	0.1681	0.0825
resistive to reactive jamming	no	no	yes
pre-sharing keys	yes	no	no

## 5 Conclusion and future work

The design of chaos-based hop selection is a new development that offers improved performance in transmission of information without pre-shared key and also increases the security in wireless communication network. The percentage of error reduction by the reactive jamming for jamming duration 0.01 and 0.05 s for FHSS and CFHSS is 55.03 and 84.24%, respectively. The obtained result shows that CFHSS technique is more secure and difficult to jam by the reactive jammer. When frequency hop is used alone, a high-throughput overhead and pre-sharing of key for channel selection is required. Similarly, to overcome jammer in rate adaptation (RA) [14, 15], the transmitter forced to operate at the lowest transmission rate if jammer exists in between two legitimate users. So when these techniques are used separately, the performance shown is to be ineffective. Therefore in future work, we will combine both CFHSS and RA technique and analyse the optimal transmission quality of data under time-varying channel conditions in secured manner.
